# Impact of COVID-19 on Economic Well-Being and Quality of Life of the Vietnamese During the National Social Distancing

**DOI:** 10.3389/fpsyg.2020.565153

**Published:** 2020-09-11

**Authors:** Bach Xuan Tran, Hien Thi Nguyen, Huong Thi Le, Carl A. Latkin, Hai Quang Pham, Linh Gia Vu, Xuan Thi Thanh Le, Thao Thanh Nguyen, Quan Thi Pham, Nhung Thi Kim Ta, Quynh Thi Nguyen, Cyrus S. H. Ho, Roger C. M. Ho

**Affiliations:** ^1^Institute for Preventive Medicine and Public Health, Hanoi Medical University, Hanoi, Vietnam; ^2^Bloomberg School of Public Health, Johns Hopkins University, Baltimore, MD, United States; ^3^Institute for Global Health Innovations, Duy Tan University, Da Nang, Vietnam; ^4^Faculty of Medicine, Duy Tan University, Da Nang, Vietnam; ^5^Center of Excellence in Evidence-based Medicine, Nguyen Tat Thanh University, Ho Chi Minh City, Vietnam; ^6^Department of Psychological Medicine, National University Hospital, Singapore, Singapore; ^7^Department of Psychological Medicine, Yong Loo Lin School of Medicine, National University of Singapore, Singapore, Singapore; ^8^Institute for Health Innovation and Technology (iHealthtech), National University of Singapore, Singapore, Singapore

**Keywords:** social distancing, economic well-being, household income, quality of life, HRQOL, COVID-19

## Abstract

Starting from April 1st, 2020, the nationwide partial lockdown in Vietnam has shown the effectiveness in stopping the community transmission of COVID-19, however, it also produced adverse impacts on the economy and inhabitants’ life. A cross-sectional study using a web-based approach was conducted in the second week of April 2020 to examine the influence of the national social distancing on the quality of life and economic well-being of Vietnamese citizens under COVID-19 pandemic. The data included socio-economic characteristics, impact of COVID-19 on household income, health status, and health-related quality of life (HRQOL). Ordered logistic regression and multivariable Tobit regression model were employed to examine factors correlated to income change and HRQOL. Results showed that among 341 participants, 66.9% reported household income loss due to the impact of COVID-19. People holding undergraduate degrees, working in other sectors rather than healthcare, and having definite-term contract had a higher likelihood of income reduction. The mean score of EQ-5D-5L and EQ-VAS was 0.95 (± 0.07) and 88.2 (± 11.0), respectively. The domain of Anxiety/Depression had the highest proportion of reporting any problems among 5 dimensions of EQ-5D-5L (38.7%). Being female, having chronic conditions and living in the family with 3–5 members were associated with lower HRQOL scores. A comprehensive assessment of the influence of COVID-19 along with public health interventions, especially mental health programs, should be implemented to mitigate the negative effects of this pandemic on the economic status and quality of life of citizens.

## Introduction

Emerging from December 2019 in Wuhan, China, coronavirus disease 2019 (COVID-19) has posed one of the greatest challenge to humankind within 75 years since World War Two (UNDP, 2020). As of July 10, 2020, there have been more than 12 million confirmed cases and 550,000 people lost their lives because of severe acute respiratory syndrome coronavirus 2 (SARS-CoV-2) (Johns Hopkins University, 2020). While a specific treatment for this disease is still lacking (Harvard Medical School, 2020), the World Health Organization recommended that every nation plan and take comprehensive public health actions to suppress the spread of COVID-19 (WHO, 2020). In addition to medical-related measures such as testing and treating for patients, most countries are implementing temporary mobility restriction, social distancing, and large-scale gathering cancelation. These strategies have shown their effectiveness in slowing the transmission speed of SARS-CoV-2, however, they also have side effects on many aspects of citizen’s lives (UNDP, 2014). The International Labor Organization estimates that 195 million jobs could be lost in the second quarter of 2020 as a result of COVID-19 (International Labour Organization, 2020). More than 1.5 billion students have been affected by the closure of educational institutions, reported by the United Nations Educational Scientific and Cultural Organization (UNESCO, 2020). People living in poverty, the elderly, youth, and indigenous persons are among the most vulnerable population in terms of suffering detrimental effects from the SARS-CoV-2 virus (UNDESA, 2020). It is imperative for every government to implement impact mitigation programs, not only interventions for healthcare or business but also consider improving both socioeconomic status and quality of life (QOL) of the general population, especially the vulnerable groups.

A pandemic can cause economic disruption in different ways. Human behavioral changes, such as fear-induced aversion to places of work and public gatherings, are a major cause of economic damage, besides the impact of mitigation measures (Madhav et al., 2017). Previous literature has documented the negative effects of different pandemics on the family income of residents. Wuqi Qui et al. conducted a case comparison study in China and concluded that the 2003 Severe acute respiratory syndrome (SARS) epidemic caused the average annual revenue of households to fall to US$ 175.44, 22.36% below what was anticipated, whereas the H7N9 pandemic had a less severe impact on the economy in comparison with SARS (Qiu et al., 2018). The Ebola epidemic, which mostly occurred in Africa, was reported to cause massive income deficits in numerous countries of this continent. Within just 6 months, this disease had resulted in a significant amount of household income loss in Liberia (35.13%) and Sierra Leone (29.67%) (UNDP, 2014) with the rates of reporting a decline in family income of 66% (Mercy Corps, 2019) and 55.5% (Michael et al., 2017). In terms of COVID-19 impact on household income, a recent survey in late March 2020 with 7,005 respondents from G7 countries has indicated that 31% of participants reported that “Coronavirus has already impacted my household income” and 39% thought that “Coronavirus has not yet impacted my household income, but I expect it to in the future” (Duffin, 2020). While in India, the proportion of people reporting a decline in income escalated from 9% in late February to 45.6% in mid-April 2020 (Keelery, 2020). However, the magnitude of the economic loss for each household due to SARS-CoV-2 has not been fully understood; therefore, more studies are needed to fill this gap.

In addition to causing severe consequences on the economy, COVID-19 also produced negative effects on the quality of life of residents, yet there are few studies on this topic and they only focus on a specific subject, not the general public. A study in Italy using a questionnaire to measure Health-Related Quality of Life (HRQOL) of Adults with Common Variable Immune Deficiency (CVID_QOL) has illustrated that COVID-19 disease generated negative effects on health-related quality of life and the risk of anxiety/depression among Primary Antibody Deficiencies patients (Pulvirenti et al., 2020). Utilizing 36-Item Short Form Survey (SF-36) to determine the influence of COVID-19 on health-related quality of life among people with suspected COVID-19 symptoms (S-COVID-19-S) from 5 provinces of Vietnam, Hoang et al. concluded that participants with S-COVID-19-S had a lower HRQOL-score (B, −7.92; *p* < 0.001) than those without S-COVID-19-S (Nguyen et al., 2020). To date, there is a lack of study using the standardized toolkit for measuring the impact of SARS-CoV-2 on QOL of the general population.

Vietnam is among the most vulnerable countries to COVID-19 on both economic and health aspects. The Vietnamese economy heavily relies on China as China is currently the second largest export market of this country and production in Vietnam always depends on imported raw materials, primarily from China (VietnamCredit, 2019). On health perspective, because of sharing a long border with China and limited healthcare resources, Vietnam had a higher likelihood of having COVID-19 imported cases as well as high risk of large-scale community transmission (VietnamCredit, 2019; Ebbighausen, 2020; Fleming, 2020). Although having a small number of confirmed cases and zero deaths related to COVID-19, the Vietnamese government decided to impose the nationwide partial lockdown at the early stage, starting from April 1st, 2020. This strategy has shown the effectiveness in stopping the spread of COVID-19 (Vu and Tran, 2020), however, it is freezing the economy and producing adverse impacts on inhabitants’ life. This study targets to provide empirical evidence about the influence of this national social distancing on quality of life and household income of Vietnamese citizens avid COVID-19, with the ultimate goal to inform the policymakers to take appropriate and timely actions for controlling the disease while ensuring both health and socioeconomic wellbeing of the general population.

## Materials and Methods

### Study Settings and Participants

A cross-sectional study using a web-based approach on qualified, well-known SurveyMonkey platform was conducted from April 6–12, 2020, 1 week after the first national partial lockdown was imposed by the government of Vietnam. During this time interval, all Vietnamese people were told to stay indoors and could only go out for essential services, such as buying food, medicine, and other emergency case. The citizens were required to maintain a two-meter distance between individuals; gathering in groups of more than two people in public places were prohibited; and all non-essential business were shut down (VOV, 2020). This maximum social distancing strategy was implemented in the second stage of fighting COVID-19 in this country with most of the cases imported from overseas and community transmission emerged (Le, 2020; Manh and Dinh, 2020). The inclusion criteria of respondents in our study including (1) Agreeing to participate in the study by approving the online consent form; (2) Having the ability to access the online questionnaire; (3) Having full capacity to answer the questions.

### Sample and Sampling

Participants were recruited by snowball sampling technique. Initially, a core group of 15 members including lecturers, students, and staff from Hanoi Medical University was set up. These persons had a higher probability of knowing other individuals from other medical universities and health facilities in Vietnam. This group was selected to reflect the diversity of research subjects, for example age, gender, educational level, and occupation throughout the country. Core-group members accessed the survey link via their computers, tablets, or smartphones and shared research invitation to their relatives and friends through email or social media. After people completed the questionnaire, they were encouraged to spread the link of the online survey to invite others residing in all regions of Vietnam to join. A total of 341 respondents involved in our study after 1 week of collecting data.

### Measure and Instruments

A structured questionnaire was developed based on the validated tools and piloted on lecturers and students of Hanoi Medical University prior to the data collection throughout Vietnam. The information collected from this survey included:

### Socio-Economic Characteristics

Socio-economic data included age, gender, living area, marital status, living area, religion, family size, educational level, occupation, employment status, and having health insurance.

### Impact of COVID-19 on Household Income

The impact of COVID-19 on household income was examined by two questions: (1) “How has your family’s monthly income changed due to the impact of COVID-19?” with three answer options (a) Decreased; (b) Increased; (c) Unchanged; (2) “Please estimate the percentage of your family income’s change compared with the month before COVID-19 started to occur in Vietnam?” with six choices of answering: (a) 0%; (b) < 20%; (c) 20–40%; (d) 40–60%; (e) 60–80%; and (f) 80–100%. People were classified in “changed income” if they reported their income as “decreased” or “increased.” Respondents were classified “no income change” if they replied their income “unchanged” under the impact of COVID-19.

### Influence of COVID-19 on Participant’s Job

Respondents also reported the influence of this pandemic on their jobs, which is classified in 4 options including (1) Fired; (2) Reduce working hours/shifts; (3) Have to work overtime; and (4) No effect.

### Health Status and Service Utilization

The health condition of participants was investigated based on 4 criteria: Having outpatient examinations in the last 14 days; Having COVID-19 test in the last 14 days; Being isolated in the last 14 days; Having chronic conditions.

### Health-Related Quality of Life

In this study, we chose the EuroQoL 5 Dimensions 5 Levels (EQ-5D-5L), which is a short, simple, validated questionnaire, and widely used in Vietnam for measuring the HRQOL of the general population. This tool comprises of five domains including Mobility, Self-care, Usual Activities, Pain/Discomfort, and Anxiety/Depression, which are assessed under 5 levels of answer from no problems (code 1) to extreme problems (code 5). The responses of each dimension were incorporated to determine the health state of participants with 11111 representing the best health and 55555 meaning the worst health. Each health state interpreted in one single “utility” score, which can be converted by using the interim scoring for the EQ-5D-5L (EuroQol Research Foundation, 2019). In the present study, we utilized the Vietnamese value set with the score ranging from -0.5115 to 1 (Mai et al., 2020). In addition, another element of EQ-5D-5L is Euroqol Visual Analogue Scale (EQ-VAS), was also used for participants to self-rate their health with the value ranging from 0 (worst imaginable health) to 100 (best imaginable health) (EuroQol Research Foundation, 2019). The recall period for both EQ-5D-5L and EQ-VAS was “today” – the day that respondents answered the online questionnaire.

### Statistical Analysis

The extracted data from the online survey were analyzed by STATA 15.0 software. We described data characteristics that covered frequency, percent, mean, and standard deviation. To compare differences between people having household income change due to the impact of COVID-19 and people who were not affected, we used *t*-test or Mann Whitney test for ordinal variables and Fisher-exact test or Chi-square test for nominal variables. Ordered logistic regression was employed to examine factors correlated with the impact of COVID-19 on respondents’ family income. A multivariable Tobit regression model was applied to identify factors associated with HRQOL score. A forward stepwise strategy was applied to obtain the final regression model with a threshold of the p-value is less than 0.2. A p-value of less than 0.05 was considered as statistical significance.

### Ethical Consideration

The Institutional Review Board of Institute for Preventive Medicine and Public Health, Hanoi Medical University, approved the protocol of this study (Code. QD 75/QD-YHDP&YHDP dated 27 March 2020). The research’ objectives and informed consent were provided on the online survey at the first page before the respondents make a decision to participate or not. No incentives were provided to participants and they could withdraw from the survey at any time. The respondents’ information was kept confidentially and was only for research purposes.

## Results

Of 341 participants with a mean age of 33.7, the majority of them were female (65.7%), living in Northern Vietnam (76.8%) with a family size of 3–5 people (70.7%), no religion (85%), holding undergraduate degrees (54%), having full-time jobs (62.8%) and having health insurance (96.2%). In terms of occupation, students registered the highest proportion (24.6%), followed by white-collar workers (20.8%) and health professionals (19.4%) (Table 1).

**TABLE 1:** Socio-economics characteristics of respondents.

Characteristics	Income changes due to the impact of COVID-19				Total		*p*-value
	Yes		No				
	*n*	%	*n*	%	*n*	%	
**Total**	229	67.2	112	32.8	341	100.0	
**Gender**							
Male	72	31.4	45	40.2	117	34.3	0.11
Female	157	68.6	67	59.8	224	65.7	
**Region**							
Northern	173	75.6	89	79.5	262	76.8	0.06
Central	32	14.0	8	7.1	40	11.7	
Southern	19	8.3	15	13.4	34	10.0	
Foreign	5	2.2	0	0.0	5	1.5	
**Age group**							
Under 25	58	25.3	27	24.1	85	24.9	0.99
25–34	68	29.7	34	30.4	102	29.9	
35–44	62	27.1	32	28.6	94	27.6	
Above 44	41	17.9	19	17.0	60	17.6	
**Religion**							
Yes	36	15.7	15	13.4	51	15.0	0.57
No	193	84.3	97	86.6	290	85.0	
**Marital status**							
Single	82	35.8	45	40.2	127	37.2	0.38
Living with spouse	140	61.1	66	58.9	206	60.4	
Others	7	3.1	1	0.9	8	2.4	
**Family size**							
1–2 people	41	17.9	23	20.5	64	18.8	0.36
3–5 people	167	72.9	74	66.1	241	70.7	
Above 5 people	21	9.2	15	13.4	36	10.6	
**Educational level**							
High school and below	44	19.2	31	27.7	75	22.0	0.11
Undergraduate	132	57.6	52	46.4	184	54.0	
Postgraduate	53	23.1	29	25.9	82	24.1	
**Occupation**							
Health workers	47	20.5	19	17.0	66	19.4	0.15
Professional educators	33	14.4	23	20.5	56	16.4	
White-collar workers	44	19.2	27	24.1	71	20.8	
Students	55	24.0	29	25.9	84	24.6	
Others	50	21.8	14	12.5	64	18.8	
**Employment status**							
Government officials	74	32.3	40	35.7	114	33.4	0.76
Indefinite-term contract	43	18.8	23	20.5	66	19.4	
Definite-term contract	24	10.5	10	8.9	34	10.0	
Farmers / Students / Homemakers / Unemployed / Retired	70	30.6	34	30.4	104	30.5	
Others	18	7.9	5	4.5	23	6.7	
**Having health insurance**							
Yes	217	94.8	111	99.1	328	96.2	0.05
No	12	5.2	1	0.9	13	3.8	

	***Mean***	***SD***	***Mean***	***SD***	***Mean***	***SD***	***p-value***

Age	33.4	10.3	34.2	11.9	33.7	10.8	0.88

Table 2 shows that most of the respondents reported a decline in household income (66.9%), and only one person replied that there was an increase in family income because of COVID-19. Among those having a decreased income, the change level at under 20% accounted for the highest proportion (25.2%), and the lowest went to 80–100% (6.2%). More than half of people (52.5%) answered that COVID-19 had no impact on their occupational status, while 30.2% had a reduction in working hours/shifts, and 8.5% lost their jobs as a result of SARS-CoV-2.

**TABLE 2:** Impact of COVID-19 on economic status of respondents.

Impact of COVID-19 on economic status	*n*	%
**Magnitude of household income change**		
Decreased 80–100%	21	6.2
Decreased 60–80%	23	6.7
Decreased 40–60%	52	15.3
Decreased 20–40%	46	13.5
Decreased < 20%	86	25.2
Decreased 80–100%	21	6.2
Unchanged	112	32.8
Increased	1	0.3
**COVID-19 impact on jobs**		
Fired	29	8.5
Reduced working hours/shifts	103	30.2
Have to work overtime	30	8.8
No effect	179	52.5

It can be seen from Table 3 that in the last 14 days, only 4.4% of participants reported outpatient examinations, 2.4% SARS-CoV-2 testing, and 7% being separated from the other people. There were 14.9% of respondents living with chronic diseases, including hypertension (3.0%), diabetes (1.5%), and others. The mean score of EQ-5D and EQ-VAS was 0.95 (± 0.07) and 88.2 (± 11.0), respectively. Participants with family income changed due to COVID-19 had a significantly lower score of EQ-5D (0.94 ± 0.07) than the remaining group (0.96 ± 0.6) (*p* = 0.04). Among 5 dimensions of the EQ-5D-5L scale, the aspect with the highest proportion of having any problems was Anxiety/Depression (38.7%), followed by Pain/Discomfort (10.0%), Usual activities (8.5%), while the lowest percentage went to Self-care (0.3%). A significant difference was found in the rate of having any problems in Anxiety/Depression between people with household income changed because of COVID-19 (43.7%) and those with no income change (28.6%) (*p* < 0.05).

**TABLE 3:** Health status and quality of life among respondents.

Characteristics	Household income changes due to the impact of COVID-19				Total		*p*-value
	Yes		No				
	*n*	%	*n*	%	*n*	%	
Having outpatient examination in the last 14 days	9	3.9	6	5.4	15	4.4	0.58
Having COVID-19 test in the last 14 days	7	3.1	1	0.9	8	2.4	0.28
Being isolated in the last 14 days	18	7.9	6	5.4	24	7.0	0.40
Having chronic diseases	40	17.7	10	9.2	50	14.9	0.04
Hypertension	7	3.1	3	2.8	10	3.0	0.99
Cardiovascular	0	0.0	1	0.9	1	0.3	0.33
Diabetes	3	1.3	2	1.8	5	1.5	0.66
Asthma	2	0.9	1	0.9	3	0.9	0.99
Others	29	12.8	4	3.7	33	9.9	0.01
**Mobility**							
Having problems	13	5.7	12	10.8	25	7.4	0.12
No problems	216	94.3	100	89.3	316	92.7	
**Self-care**							
Having problems	1	0.4	0	0	1	0.3	0.99
No problems	228	99.6	112	100	340	99.7	
**Usual activities**							
Having problems	23	10.1	6	5.4	29	8.6	0.21
No problems	206	90	106	94.6	312	91.5	
**Pain/discomfort**							
Having problems	26	11.3	8	7.1	34	10	0.25
No problems	203	88.7	104	92.9	307	90	
**Anxiety/depression**							
Having problems	100	43.6	32	28.6	132	38.7	< 0.01
No problems	129	56.3	80	71.4	209	61.3	

	***Mean***	***SD***	***Mean***	***SD***	***Mean***	***SD***	***p*-value**

EQ-5D index	0.94	0.07	0.96	0.06	0.95	0.07	0.04
EQ-VAS	88.3	10.1	88.0	12.6	88.2	11.0	0.64

Table 4 indicated some associated factors with the change of household income and quality of life among participants due to COVID-19. The family income of people having health insurance (OR = 0.23; 95% CI = 0.08; 0.66) and being 25 years and above were less likely to be affected, whereas persons with undergraduate degree (OR = 2.31; 95% CI = 1.17; 4.54), working in other sectors rather than healthcare (OR = 2.23; 95% CI = 1.31; 3.79), and having definite-term contract (OR = 2.13; 95% CI = 1.08; 4.19) had a higher likelihood of income reduction. Participants having chronic diseases were significantly associated with lower quality of life in both EQ-5D index (Coefficient = −0.07; 95% CI = −0.11; −0.03) and EQ-VAS (Coefficient = −6.94; 95% CI = −10.84; −3.04). Respondents being female (Coefficient = −3.36; 95% CI = −6.08; −0.04) and living with family of 3–5 people (Coefficient = −3.06; 95% CI = -6.08; −0.04) had a lower QOL likelihood in terms of EQ-VAS scale.

**TABLE 4:** Associated factors with household income change and quality of life of respondents.

Characteristics	Change of household income because of COVID-19		EQ-5D index		EQ-VAS	
	OR	95% CI	Coef.	95% CI	Coef.	95% CI
Gender (Female vs. male)					−3.36**	−6.30; −0.42
Region (Central vs. Northern)					−3.09	−7.42; 1.23
Age group (vs. Under 25)						
25–34	0.33***	0.16; 0.66				
35–44	0.38***	0.18; 0.79				
Above 44	0.35***	0.16; 0.76			3.05	−0.70; 6.80
Religion (Yes vs. no)			0.04	−0.01; 0.08		
Having health insurance (Yes vs. no)	0.23***	0.08; 0.66	0.05	−0.02; 0.13		
Having chronic diseases (Yes vs. no)			−0.07***	−0.11; −0.03	−6.94***	−10.84; −3.04
Family size (vs. 1–2 people)						
3–5 people					−3.06**	−6.08; −0.04
Above 5 people			0.04	−0.02; 0.09		
Education level (High school and below)						
Undergraduate	2.31**	1.17; 4.54				
Postgraduate	1.85	0.84; 4.08				
Occupation (Others vs. health workers)	2.23***	1.31; 3.79				
Occupation status (vs. Government officials)						
Definite-term contract	2.13**	1.08; 4.19	−0.05**	−0.10; −0.00	−4.46	−8.99; 0.08
Others					4.43	−1.07; 9.92

*** p < 0.01, ** p < 0.05.

## Discussion

Our study featured a high rate of household income loss as well as impairment on some quality of life domains among the general population in Vietnam due to the impact of the COVID-19 pandemic. We also found some potential factors associated with the change in family earnings and QOL of Vietnamese citizens, implying for future interventions and programs to enhance the socioeconomic status and well-being of the residents suffering from the epidemic in resource-constrained settings.

The findings of this research showed that more than two-thirds (66.9%) of Vietnamese participants reporting a reduction in their family income as a result of COVID-19. This figure is higher than those in India (45.6%) (Keelery, 2020) and G7 countries (31%) (Duffin, 2020). The disparity could be attributable to the difference in economic structures and major markets between countries. The economy of Vietnam heavily relied on exports, whereas China is the second largest export destination and the main consumer of agriculture products of this country (B&Company, 2020; The Observatory of Economic Complexity, 2020). The magnitude of income deficit due to COVID-19 is the new contribution of our study to enrich the evidence related to the influence of SARS-CoV-2 on the economic status of the general public. Based on the assessment of household revenue damage stated by all respondents, we understand that this disease caused disparate impacts on income, calling for specific strategies to recover the economic status of targeted groups. The previous literature only mentioned the effect of pandemics under the perspective of average income loss proportion among all participants, such as the 2003 SARS epidemic in China with the average annual income of households to fall 22.36%) (Qiu et al., 2018), the Ebola in African countries caused the deficit in 6-month household income in Liberia and Sierra Leone of 35.13 and 29.67%, respectively (UNDP, 2014). Hence, our study suggested a new direction for future research, which is discovering the influence magnitude of pandemics on the economic well-being of the general population.

In the current study, the family income of people holding undergraduate degrees, working in other sectors rather than healthcare, and having definite-term contracts had a higher likelihood of being changed as a result of COVID-19. This may be due to the downsizing or closure of businesses following the requirement of strict social distancing in Vietnam. According to the estimation by the General Statistics Office of Vietnam, as of mid-April 2020, nearly 5 million people have lost their jobs because of this epidemic (Nguyen, 2020). The most affected sectors are tourism, services, aviation, transportation, leather, and footwear (Trong Quynh, 2020). To mitigate the economic impact of this pandemic, on April 10th, 2020, the Prime Minister of Vietnam has approved the relief packet of US $2.6 billion, targeting about 4.135 million victims who are hardest hit by COVID-19 (Minh, 2020; Vietnam News, 2020). However, the main beneficiaries of this policy are mostly the poor and near-poor, people with records of meritorious services to the country, household businesses, employers, freelance labors and blue-collar workers, and the list of recipients only based on the reports from local authorities (Prime Minister, 2020), while employees with higher educational degrees seemed to be neglected. Scientific evidence is still lacking to advise this policy and our research was one of the first studies to contribute important information for decision-makers to consider their actions. As our finding had pointed out, the income of employees having advanced education even more affected than those only have high school diplomas or lower, therefore, the policymakers should develop appropriate strategies to reduce the inequality in accessing financial support during the time of COVID-19.

Regarding the quality of life aspect, the average score of EQ-5D index (0.9) and EQ-VAS (88.2) calculated from our study were consistent with previous research among the general population of Vietnam in the context without the influence of pandemic (0.91 and 87.4, respectively), however, the rate of having Anxiety/Depression in this study (38.7%) was much higher than that in the compared research (15.2%) (Nguyen et al., 2017). Banned outdoor recreation, fear of being infected SARS-COV-2, and concerns of impact of this disease might be the reasons leading to a higher proportion of anxiety/depression in our study. Other studies in China also emphasized the high prevalence of mental health disorders among the general public under the influence of COVID-19 (Huang and Zhao, 2020; Ni et al., 2020; Wang C. et al., 2020; Wang Y. et al., 2020b). This high rate calls for the implementation of mental health strategies to minimize the negative impact of this disease on the mental health of residents (Ho et al., 2020). On the other hand, our analysis reveals that those being women and having chronic conditions were found to have lower HRQOL scores, which was in line with previous studies (Ha et al., 2014; Nguyen et al., 2017, 2019; Pham et al., 2018). The participants living in a family with 3–5 members had a higher risk of impaired QOL than those in households with a smaller size. The possible explanation is that during the nationwide partial lockdown, people were forced to stay at home and had no income to pay for living expenses; thus, the more people in the family, the higher the financial burden.

Several implications can be derived from our study. First, as our study provided preliminary findings at the early stage of national partial lockdown in Vietnam, it is critically necessary to have a comprehensive assessment on the effect of COVID-19 among different subjects in the next periods which can inform the government on policies for easing economic pain of the most affected populations. Second, public health interventions, especially mental health programs, should be implemented to address the psychological impacts of this pandemic on the quality of life of the general population. Finally, the evidence from our study can contribute to the development of more effective preparedness and response strategies to diminish the impact of future pandemics in Vietnam and other low- and middle-income countries.

The interpretations of the findings from our study should consider the following limitations. Since the authors did not collect the information on how many people the respondents have invited to participate in this survey, and it is possible that several individuals may invite the same person, we were not able to know exactly the number of people actually invited and the response rate in this online survey. The nature of the cross-sectional study restricts the ability to draw the causal relationship between the change in household income, quality of life, and their associated factors. The online survey could be seen as one of the best methods to collect data from a large sample size with low cost and time-saving, however, the disadvantages of this approach are possible bias of self-report and high homogeneity of data as no strict supervision is performed. Another consideration is the small sample size, which limits the generalizability for the other populations. Despite the above restraints, our research has some strengths that should be acknowledged. In the context of the national lockdown to be implemented for the first time in Vietnam, this paper provided up-to-the-minute evidence for the Vietnamese government and other low-resource settings to develop timely strategies to minimize the impact of COVID-19 pandemic on socioeconomic status and well-being of their citizens. Moreover, utilizing standard tools (EQ-5D, EQ-VAS) to measure the quality of life of people has increased the validity of this study.

In conclusion, this study depicted a high rate of household income loss as well as impairment on some quality of life domains among the general population in Vietnam due to the impact of COVID-19. It is critically necessary to have a comprehensive assessment of the effect of COVID-19 among different subjects in the next periods, which can inform the government to impose suitable policies for easing economic pain of the most affected populations. Public health interventions, especially mental health programs, should be implemented to address the psychological impacts of this pandemic on the quality of life of citizens.

## Data Availability Statement

The raw data supporting the conclusions of this article will be made available by the authors, without undue reservation, to any qualified researcher.

## Ethics Statement

The studies involving human participants were reviewed and approved by the Institutional Review Board of Institute for Preventive Medicine and Public Health, Hanoi Medical University. The patients/participants provided their written informed consent to participate in this study.

## Author Contributions

BT, HN, HL, and RH: conceptualization. TN, QP, NT, and QN: data curation. HP, HN, and LV: formal analysis. BT, HN, CL, and CH: methodology. HL and XL: supervision. BT, CL, and RH: validation. BT, HN, HP, and LV: writing – original draft. HL, CL, XL, TN, QP, NT, QN, CH, and RH: writing – review and editing. All authors have read and approved the final version of the manuscript.

## Conflict of Interest

The authors declare that the research was conducted in the absence of any commercial or financial relationships that could be construed as a potential conflict of interest.
